# Airway Management: why training, judgement, and competence remain the most important technologies

**DOI:** 10.1016/j.bjane.2026.844781

**Published:** 2026-06-15

**Authors:** Daniel Perin, Rodrigo Leal Alves

**Affiliations:** a Hospital Israelita Albert Einstein, São Paulo, SP, Brazil; b Universidade Federal da Bahia (UFBA), Salvador, BA, Brazil; c Universidade Estadual Paulista (UNESP), São Paulo, SP, Brazil; d Hospital São Rafael, Salvador, BA, Brazil

Airway management has undergone a remarkable transformation over the past two decades. Better data on safety and efficacy of different techniques and treatment algorithms, the expansion of video laryngoscopy, the evolution of second-generation supraglottic airway devices (SADs), the emergence of point-of-care ultrasound and the development of low-cost technological innovations have substantially increased the number of tools available to anesthesiologists. Yet, despite these advances, the determinants of successful airway management remain fundamentally unchanged: knowledge, training, judgment, and experience.^1^^,^^2^

In this issue of the Brazilian Journal of Anesthesiology, three complementary studies provide important insights into contemporary airway practice.^3^, ^4^, ^5^ Together, they highlight a central message for the specialty: technological innovation alone is insufficient unless accompanied by structured airway education, clinical judgment, and sustained training.

The randomized trial by Mendes et al.^3^ represents an important step toward democratizing access to video laryngoscopy. The development of a reproducible, low-cost 3D-printed videolaryngoscope addresses a critical global need. The study was performed in adult patients scheduled for surgical procedures under general anesthesia and involved anesthesiologists. Although the device provided superior glottic visualization compared with conventional Macintosh laryngoscopy, improved visualization did not translate into easier or faster intubation. Despite all limitations of the study, it emphasizes the importance of device-specific training and airway expertise.

This apparent paradox is well recognized in airway management. Even though there is a robust set of evidence that video laryngoscopy offers improved visualization of vocal cords when compared with conventional direct laryngoscopy, obtaining an excellent view of the glottis does not necessarily guarantee successful tube delivery.^6^^,^^7^ Device-specific psychomotor skills, hand-eye coordination, stylet manipulation, and familiarity with indirect visualization techniques remain essential competencies. Another layer of discussion in that matter involves the scarcity of studies comparing 3D-printed models with commercially available products with little evidence favoring the latter.^8^^,^^9^

The notion that SADs have an important role not only in anesthesia but also in emergency and resuscitation scenarios is not new and has been around now for over three decades.^10^^,^^11^ Concerning that subject, Santos Neto et al.^4^ conducted a national survey that reinforced the relevance of training and skill. Although Brazilian anesthesiologists overwhelmingly perceived supraglottic airway devices as safe and effective, actual utilization remained modest. Exposure during residency emerged as one of the strongest predictors of current clinical use, while lack of operator experience was identified as the most important barrier to broader adoption.

The importance of comprehensive airway training is further underscored by the retrospective cohort study by Azem and colleagues.^5^ Examining patients undergoing surgical drainage of severe odontogenic infections, the authors demonstrated that Awake Fiberoptic Intubation was preferentially selected for anatomically complex cases characterized by trismus and overweight patients with elevated body mass index. Importantly, the study illustrates that successful airway management in high-risk patients depends not only on access to advanced devices but also on the ability of clinicians to recognize predictors of difficulty, select the most appropriate airway strategy, and execute advanced techniques safely. The finding that postoperative spontaneous ventilation was associated with earlier discharge further highlights how airway management decisions can influence the broader perioperative trajectory.

Together with the studies on video laryngoscopy and supraglottic airway devices, the collected data emphasize that airway excellence is built upon a hierarchy of competencies ranging from routine device use to the management of the most challenging anticipated difficult airways. Recently, international literature has further strengthened this perspective. Contemporary discussions on airway education have argued that modern airway management should be viewed as a competency continuum rather than a collection of isolated technical skills. The focus is shifting from simply mastering devices toward developing integrated airway expertise, including decision-making, team communication, crisis resource management, and adaptability in rapidly evolving clinical situations.^1^

Collectively, the studies presented in this issue illustrate two sides of the same coin. Innovation is expanding access to airway technologies, yet even widely accepted technologies remain underutilized when training opportunities are insufficient. The challenge facing anesthesiology is therefore not merely technological dissemination but educational implementation.

For Brazil and other resource-variable healthcare systems, these findings are particularly relevant. Investments in airway education, simulation programs, residency curricula, and continuing professional development may ultimately have a greater impact on patient outcomes than the acquisition of additional devices alone. The future of airway management will undoubtedly include increasingly sophisticated technologies. However, the most important innovation may not be a new device but rather the development of robust educational ecosystems capable of transforming technology into clinical competence.

As the studies by Mendes et al., Santos Neto et al., and Azem et al. demonstrate airway safety is achieved not when clinicians have access to more devices, but when they are trained to use the right device, at the right time, for the right patient. In airway management, training, judgment, and competence remain the most important technologies.

## Conflicts of interest

The authors declare no conflicts of interest.
